# Diagnostic Accuracy of MRI in Detecting the Perineural Spread of Head and Neck Tumors: A Systematic Review and Meta-Analysis

**DOI:** 10.3390/diagnostics14010113

**Published:** 2024-01-04

**Authors:** Umida Abdullaeva, Bernd Pape, Jussi Hirvonen

**Affiliations:** 1 Department of Radiology, Tashkent City Branch of the Republican Specialized Scientific and Practical Medical Center of Oncology and Radiology, Tashkent 100054, Uzbekistan; 2 Department of Biostatistics, University of Turku and Turku University Hospital, 20521 Turku, Finland; bernd.pape@tyks.fi; 3 School of Technology and Innovations, University of Vaasa, 65101 Vaasa, Finland; 4 Department of Radiology, Tampere University Hospital and Tampere University, Faculty of Medicine and Health Technology, 33100 Tampere, Finland; jussi.hirvonen@utu.fi

**Keywords:** cranial nerves, head and neck tumors, perineural spread, magnetic resonance imaging, systematic review, meta-analysis

## Abstract

The purpose of this study was to review the diagnostic accuracy of MRI in detecting perineural spreading (PNS) of head and neck tumors using histopathological or surgical evidence from the afflicted nerve as the reference standard. Previous studies in the English language published in the last 30 years were searched from PubMed and Embase databases. We included studies that used magnetic resonance imaging (MRI) (with and without contrast enhancement) to detect PNS, as well as the histological or surgical confirmation of PNS, and that reported the exact numbers of patients required for assessing diagnostic accuracy. The outcome measures were sensitivity, specificity, positive predictive value (PPV), and negative predictive value (NPV). Heterogeneity was assessed with the Higgins inconsistency test (I^2^). P-values smaller than 0.05 were considered statistically significant. A total of 11 retrospective studies were found, reporting 319 nerve samples from 245 patients. Meta-analytic estimates and their 95% confidence intervals were as follows: sensitivity 0.85 (0.70–0.95), specificity 0.85 (0.80–0.89), PPV 0.86 (0.70–0.94), and NPV 0.85 (0.71–0.93). We found statistically significant heterogeneity for sensitivity (I^2^ = 72%, *p* = 0.003) and PPV (I^2^ = 70%, *p* = 0.038), but not for NPV (I^2^ = 65%, *p* = 0.119) or specificity (I^2^ = 12%, *p* = 0.842). The most frequent MRI features of PNS were nerve enlargement and enhancement. Squamous cell carcinoma and adenoid cystic carcinoma were the most common tumor types, and the facial and trigeminal nerves were the most commonly affected nerves in PNS. Only a few studies provided examples of false MRI diagnoses. MRI demonstrated high diagnostic accuracy in depicting PNS of cranial nerves, yet this statement was based on scarce and heterogeneous evidence.

## 1. Introduction

Perineural spread (PNS) is defined as tumor extension beyond the site of origin to distant locations along nerves and neural sheaths [1,2], and it can occur independently from other types of tumor spread [3]. The PNS of head and neck tumors is associated with a worse prognosis, increased local recurrence rates, and aggressiveness [4,5]. In advanced stages, the disease can cause neuropathic pain and functional impairment of the affected cranial nerves (CNs), leading to significant morbidity [6]. PNS is most common in adenoid cystic carcinoma (ACC) of the salivary glands and in squamous cell carcinoma (SCC), with reported prevalences of up to 56% and 34%, respectively, followed by mucoepidermoid carcinoma, desmoplastic melanoma, lymphoma, and sarcoma [2,3,7,8,9,10,11]. Because of the extensive innervation in the head and neck region, trigeminal and facial nerves are most often afflicted by PNS [3,5,9,12].

About 40–45% of patients in the early stage of PNS may be clinically silent. However, treatment at this stage has a high cure rate [3,4]. Insufficient clinical and radiological awareness of specialists, as well as inadequate skull base imaging of PNS, can result in delayed diagnosis and treatment [6,13]. Hence, the evaluation of the early stage of PNS of the tumor on imaging is vital for the staging and prognosis of the disease [7,13,14]. Among the imaging methods, magnetic resonance imaging (MRI) is considered the overall best method for assessing PNS because of its high soft tissue contrast, multiplanar capability, and accuracy in determining the presence and extent of nerve involvement [3,9]. Studies have shown that MRI is superior to computed tomography (CT) in assessing Vidian nerve PNS [14], intracranial spread of the nasopharyngeal carcinoma [15], or spreading tumors to the skull base [16]. The superiority of MRI over CT is due to higher soft tissue contrast, and CT imaging often provides only indirect signs of PNS (such as widening or erosion of skull base foramina). Hybrid imaging is a useful technique that provides valuable insights into monitoring post-treatment and advanced disease in head and neck tumors [3]. For example, positron emission tomography/computed tomography (PET/CT) with the glucose analog [18F]FDG is highly sensitive in detecting PNS [17], although false negative and false positive results occur [18].

The diagnostic accuracy of MRI in detecting PNS varies among studies. Although in some studies, MRI showed high sensitivity and specificity in determining PNS [19,20,21], sampling bias could influence the false negative and false positive results and diagnostic accuracy of the studies. PNS must be differentiated from reactive or inflammatory nerve swelling and radiation-induced neuritis [10,21]. If only histologically confirmed cases with PNS are included in the study, specificity cannot be precisely assessed [22]. Conversely, if only MRI-positive cases are included, only the positive predictive value (PPV) can be measured. PPV can be affected by sampling, differential and partial verification bias, small sample size, patient selection type, and different reference methods [23].

This study aimed to review the evidence for the diagnostic accuracy of MRI in detecting PNS of head and neck tumors when using histopathological or surgical evidence from the afflicted nerve as the reference standard. Such proof is required as a reference standard to avoid selection bias and circumferential reasoning. We conducted a meta-analysis to gain overall estimates of diagnostic accuracy across the studies.

## 2. Materials and Methods

We performed this systematic review and meta-analysis according to the Preferred Reporting Items for Systematic Reviews and Meta-Analyses (PRISMA) guideline [24]. The search protocol was not registered in advance. We searched for studies published in English from the PubMed and Embase databases in the last 30 years, using the following terms “perineural spread”, “MRI”, and “pathology correlation” with the AND/OR operators (Table S7). In addition, the reference papers of relevant studies were searched. We excluded case reports and duplicates.

The relevant studies were identified based on the following inclusion criteria: (1) study published in English from 1 January 1992 to 31 October 2022; (2) patients with head and neck tumor; (3) MRI (with and without contrast enhancement) suggesting PNS; (4) histological or surgical confirmation of PNS; (5) reported number of patients required for assessing diagnostic accuracy: total number of patients undergoing biopsy or surgery after MRI suggestive of PNS. We did not use any exclusion criteria.

Two radiologists (U.A., J.H.) independently screened and extracted data from the included studies. The following data were extracted from the relevant studies: authors, study year, study design, number of patients, age of patients, reference standard (histology or surgery), number of analyzed nerves, number of true and false MRI findings, primary tumor histology and location, nerves affected by PNS, MRI field strength, MRI technique and protocols, MRI contrast agent usage, and MRI features of PNS.

The quality of the included studies was assessed according to the Quality Assessment Diagnostic Accuracy Studies statement-2 (QUADAS-2) [25], which consisted of four domains (Tables S1 and S8).

For each study, we assessed true positives (TP), false positives (FP), true negatives (TN), and false negatives (FN). We then calculated estimates of sensitivity (TP/[TP+FN]), specificity (TN/[TN+FP]), PPV (TP/[TP+FP]), and negative predictive value (NPV) (TN/[TN+FP]). Meta-analytic estimates were derived using random effects modeling with Proc Mixed on SAS System, version 9.4 for Windows (SAS Institute Inc., Cary, NC, USA). We applied the random effects model by DerSimonian and Laird [26] to logit transformed sensitivity, specificity, and predictive values using the algorithm described by Normand [27]. Heterogeneity was assessed with the Higgins inconsistency test (I^2^). P-values smaller than 0.05 were considered statistically significant.

## 3. Results

### 3.1. Study Selection

**Figure 1:**
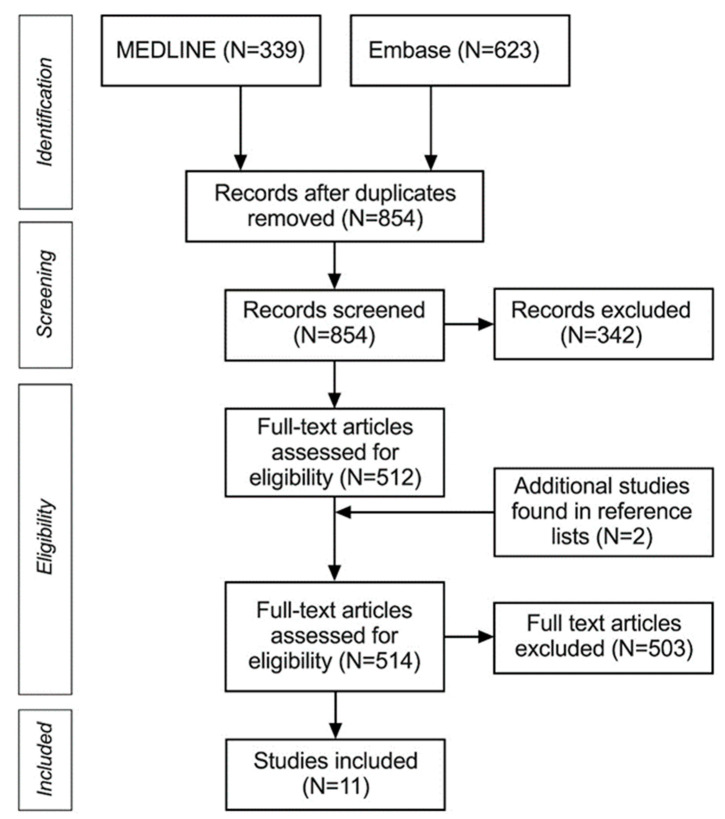
PRISMA flowchart of systematic review and meta-analysis.

As shown in Figure 1, we found 11 studies to be included in the systematic review and meta-analysis. All studies were retrospective and reported on a total of 319 nerves sampled from 245 patients (mean age 59, range 17–91). Ten studies [8,16,19,20,21,22,28,29,30,31] used histology as a reference method for CNs involved in the PNS, and only one study [32] used surgical confirmation. One study [19] included two models of the descending facial nerve (DFN), depending on the threshold of abnormality, and one model of involvement of the stylomastoid foramen (SMF); these were all included in the meta-analysis separately for completeness.

Two studies were not included in the systematic review due to a lack of precise MRI data on patients with PNS [33,34].

### 3.2. Diagnostic Performance

**Figure 2:**
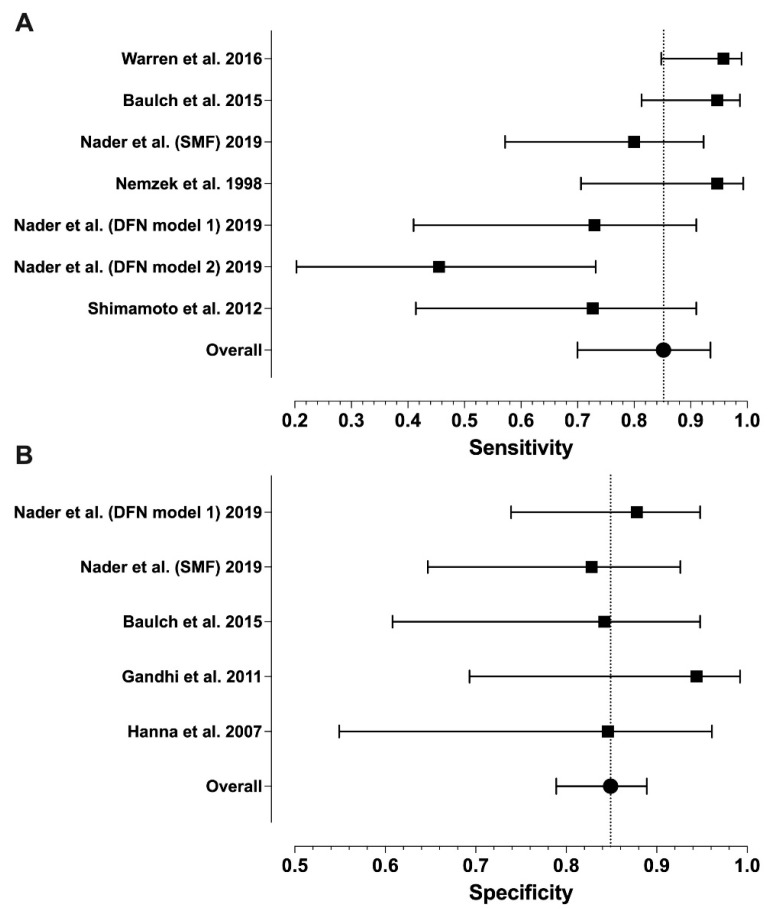
Forest plots of the sensitivity (**A**) and specificity (**B**) values from individual studies and their model-based estimates. Bars represent 95% confidence intervals, and dotted lines represent the model-based estimates. Data shown from original publications: (**A**) [19,21,22,28,30], (**B**) [16,19,20,21].

**Figure 3:**
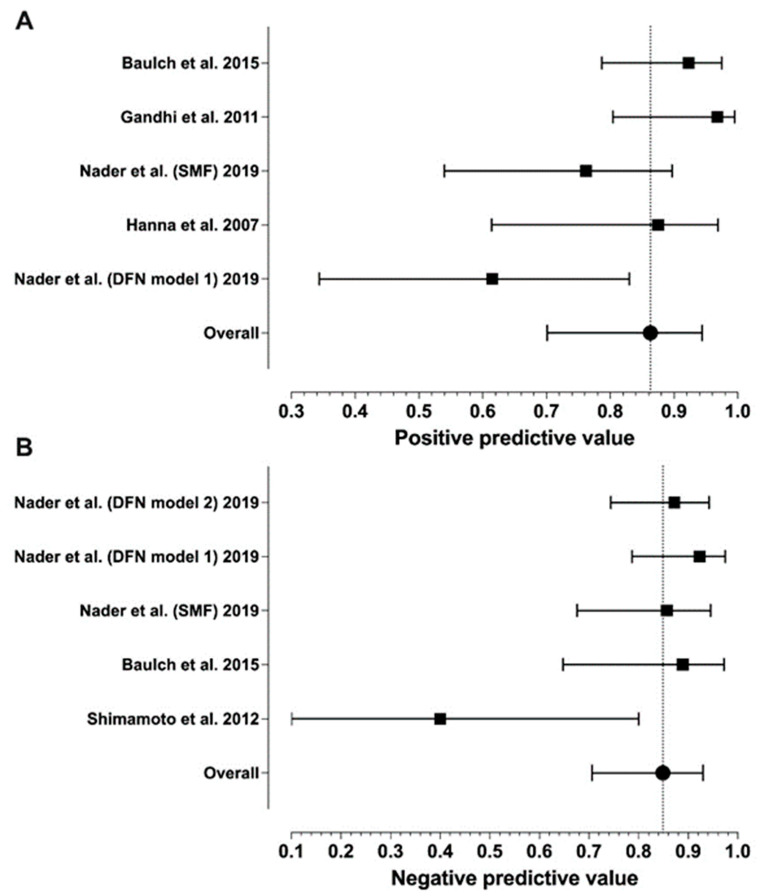
Forest plots of the PPV (**A**) and NPV (**B**) values from individual studies and their model-based estimates. Bars represent 95% confidence intervals, and the dotted lines represent the model-based estimates. Data shown from original publications: (**A**) [16,19,20,21], (**B**) [19,21,30].

Sensitivity ranged from 0.46 to 1.00 and specificity from 0.83 to 1.00, with median values of 0.96 and 0.88, respectively (Table 1). Meta-analytic estimates and their 95% confidence intervals are shown in Figure 2 and Figure 3: sensitivity 0.85 (0.70–0.95), specificity 0.85 (0.80–0.89), PPV 0.86 (0.70–0.94), and NPV 0.85 (0.71–0.93). Because of missing data (zero values in the 2 × 2 tables), only six studies were included in the sensitivity analysis and four in the specificity analysis. We found statistically significant heterogeneity for sensitivity (I^2^ = 72%, *p* = 0.003) and PPV (I^2^ = 70%, *p* = 0.038), but not for NPV (I^2^ = 65%, *p* = 0.119) or specificity (I^2^ = 12%, *p* = 0.842).

### 3.3. Quality Assessment

Risk was rated as low in all studies related to flow of timing (risk of bias), tumor types of PNS (applicability concerns, patient selection), and interpretation of pathology reports (applicability concerns, reference standard), as well as in most studies regarding the reliability of MRI (risks of bias, index test), but in other domains was high or unclear (Tables S1 and S8).

### 3.4. Primary Tumor Histology and Location

**Figure 4:**
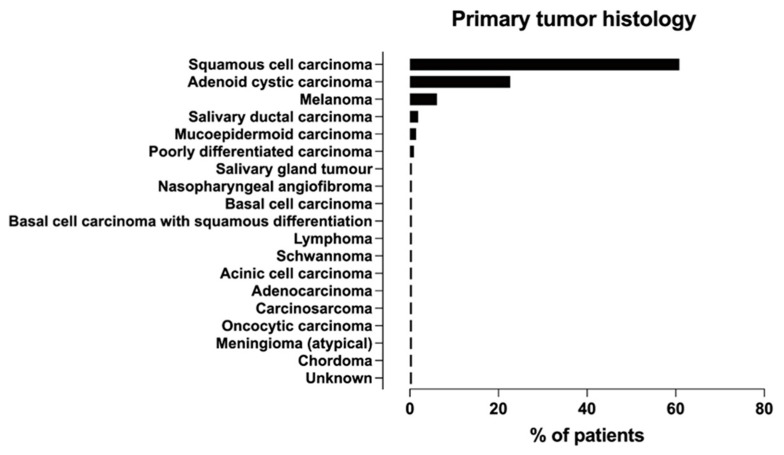
Frequency distribution of primary tumor histologies associated with PNS across studies.

All studies reported tumor histology, and eight [8,16,22,28,29,30,31,32] reported the location of head and neck tumors afflicted by PNS, with detailed numbers in five studies [8,22,28,30,31] regarding the primary site of origin (Table S2). As shown in Figure 4 and Table S6 the most common histological tumor types were SCC and ACC, followed by melanoma. PNS has rarely been studied in some tumors (such as meningioma, schwannoma, and chordoma). Only two studies have provided data on the incidence of PNS [16,19].

### 3.5. Nerves Affected by PNS

**Figure 5:**
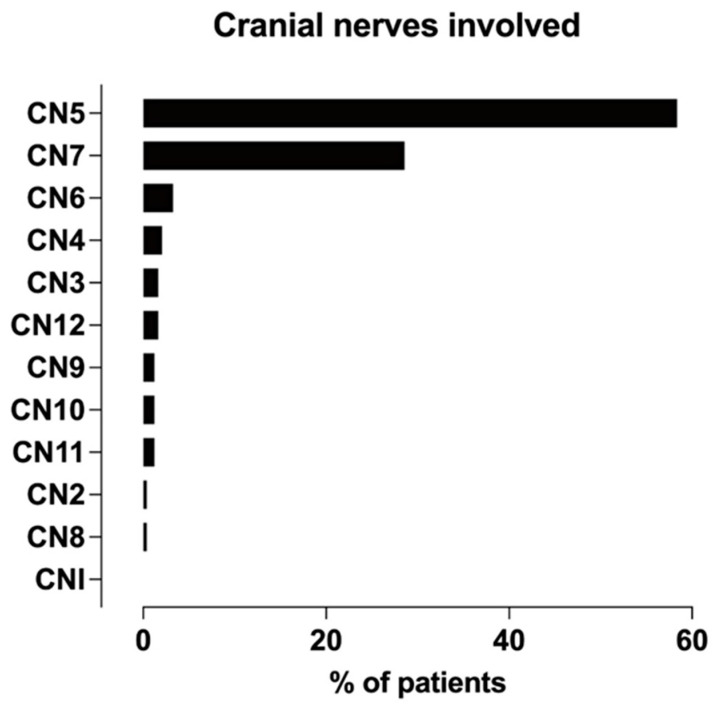
Frequency distribution of cranial nerves afflicted with PNS across studies.

Figure 5 illustrates that the trigeminal and facial nerves were most often afflicted, whereas the great auricular, optic (CN II), and vestibulocochlear nerves (CN VIII) were least often affected by PNS. Targeted MRI studies have focused on the large nerve PNS [20,21,28], whereas studies with conventional MRI protocols have examined many CNs [8,16,19,22,29,30,32]. Tomura et al. elucidated pterygopalatine fossa (PPF) obliteration in patients with tumors in the skull base with no evaluation of the CNs [31] (Tables S2 and S3).

### 3.6. MRI Technique and Protocols

A total of 36% of studies used 1.5T MRI [20,22,30,31], 18% used 3T [21,28], and 46% did not report [8,16,19,29,32] the field strength of the MRI device. Only four studies reported data on contrast agent use [22,29,30,31] and coil types [16,20,21,30], and one reported on delaying scans after contrast administration [30]. Seven studies (58%) used conventional [8,16,19,22,29,30,31] and three (25%) used targeted MRI (neurography) [20,21,28] protocols, with one study (9%) not reporting the protocol used [32]. In terms of MRI sequences, seven studies (58%) [19,20,21,22,29,30,31] reported MRI slice thickness and/or gap or field of view (FOV) (Table S3).

The most common precontrast sequences for conventional MRI protocols were axial T2 and T1 without fat suppression (FS), and for MRI neurography, axial and coronal T2 with FS and T1 without FS. Additional precontrast FS T2 [8,30] and T1 [30] MR sequences were used for the conventional MRI protocols. Studies using both conventional and targeted MR protocols in postcontrast imaging focused on axial and/or coronal T1-weighted FS images [8,16,19,20,21,22,29,30,31] (Table S4).

### 3.7. MRI Features of PNS

**Figure 6:**
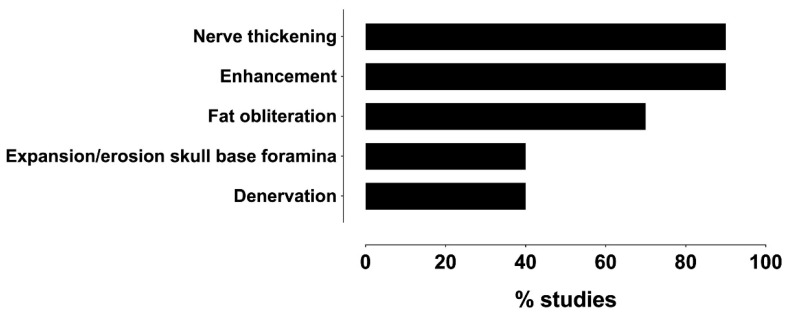
Frequency distribution of MRI features reportedly associated with PNS across studies.

Regarding the MRI findings of the PNS of the head and neck tumors, ten studies (91%) reported imaging features [8,16,19,20,21,22,29,30,31,32], and one study did not report [28] (Table S3). As shown in Figure 6, the most common PNS features on the MRI were nerve thickening and abnormal asymmetric enhancement.

## 4. Discussion

### 4.1. Diagnostic Performance

We found high accuracy for MRI in detecting PNS of head and neck tumors. The meta-analytic estimates were 0.85, 0.85, 0.86, and 0.85 for sensitivity, specificity, PPV, and NPV, respectively. However, not all studies could be included in the meta-analysis because they had some zero values in the diagnostic accuracy tables. For example, the PPV and sensitivity of many studies were 1.00 because of the absence of FP [8,22,28,29,30,31,32] and FN [8,16,20,29,31,32], respectively. These outcomes had significant heterogeneity among studies. The specificity of the six studies could not be measured because of the absence of FP and TN results [8,22,28,29,31,32]. Prospective studies with large samples are required for unbiased estimates of diagnostic accuracy.

A recent study, published after a literature search for the current systematic review and meta-analysis, used 3T MR neurography to assess the facial nerve PNS of cutaneous SCC [13]. This study found a sensitivity of 89% and a PPV of 97%, and the zonal extent was correctly identified in all TP cases. The high accuracy of 3T MR neurography is consistent with previous reports included in this review [16,19,20,21].

Partial verification bias refers to when subjects with a certain test result (MRI positive for PNS) are more likely to be confirmed histologically or surgically than those with another test result (MRI negative for PNS) [35]. Partial verification bias in studies leads to the underestimation of FN results, overestimation of sensitivity and PPV, and decreased specificity. Consecutive enrollment of patients and retrospective data collection can increase specificity, whereas the differential bias may affect study accuracy [23].

Some studies have provided evidence of FP [16,19,20,21] and FN [19,21,22,28,30]. The reported causes of FP in large nerve studies were considered to be radiation-induced neuropathy or new tumor lesions in the nerves [21], whereas in studies with conventional MRI protocols, they were associated with tumor tissue in the surrounding soft tissue of the SMF [19] or in the periosteum of the foramen ovale [22], without extension to the nerve.

MRI neurography studies had fewer FN than studies with conventional protocols. As shown in Table 1, in studies reporting FN results, the NPV ranged from 0.4 to 0.92. Since a complete 2 × 2 diagnostic table is unavailable, sensitivity and PPV will likely increase, and specificity and NPV cannot be properly measured. According to the literature, PNS can occur in asymptomatic patients [11,14], which amounts to approximately 40–45% [3,9], and can cause FN results, which can occur, especially in the earliest stages of PNS.

Prospective larger studies are necessary to obtain a true evaluation of specificity and sensitivity and an actual number of TP and FN, which requires a follow-up period [13,19,20]. Further studies should be conducted to analyze the effect of radiation-induced neuropathy in large nerve PNS results [21], as well as diagnostic variables that distinguish PNS from other neoplastic and non-neoplastic diseases.

### 4.2. Quality Assessment

We used the QUADAS-2 criteria to evaluate the quality of the studies included in the review [25]. In terms of patient selection, only three studies reported consecutive samples of selection [16,28,31], which could introduce sampling bias as it could not cover all patients without evidence of PNS. Studies included patients with head and neck tumors that underwent surgical excision [16,19,20,21,22,31] or with clinical and/or MRI evidence of PNS [8,28,29,32], which might increase imaging-positive results on MRI [28]. Patients without CN symptoms are less likely to be imaged with MRI for PNS.

In addition, studies had incomplete or different reference methods [29,31,32]. In the studies, we found partial surgical [32] (7 out of 15 cases) or histological [29,31] (only 2 out of 15 cases and 12 out of 30, respectively) confirmation of PNS. In terms of flow and timing, the recommended time between MRI and surgery was one month [21,36], but Nader et al. [19] could not find statistically significant data on the sensitivity and specificity of MRI at different time points.

In summary, the analysis of this review is restricted by many confounding factors.

### 4.3. Primary Tumor Histology and Location

Analysis of these studies highlights the difficulties in identifying the frequency of PNS among various tumors. Among the studies that evaluated PNS of ACC, its incidences were 85% [30] and 66% [16], the latter being more consistent with other studies [11,37]. However, we did not find reliable data on the incidence of PNS in other studies. Among the studies that provided data regarding tumor location afflicted by PNS, the most often affected site in a cutaneous SCC was the cheek (20%) [28]; in patients with ACC, the palate (45%) [30]; in patients with SCC, the maxillary sinus (91%) [31]; and in a small sample with melanoma, the lip (37.5%) [8]. Large nerve studies mainly included patients with SCC (81.8–100%) [20,21,28].

Overall, the evidence is not robust enough to allow for conclusive statements about potential differences in the incidence of PNS with regard to tumor histology and localization.

### 4.4. Nerves Affected by PNS

Among studies that examined the involvement of the trigeminal nerve in the PNS, the maxillary nerve was the most common division, followed by mandibular and ophthalmic divisions [8,20,21,22,28,32], consistent with the literature [7,38] (Table S5). The superficial branches of the large nerve were described in the report as difficult to detect and can lead to FN [21].

Many studies found multiple CNs afflicted by PNS [8,20,21,22,28], in 18–86% of patients, with the most common scenario being the concurrent involvement of VII and V3 in large nerve studies [20,21,28].

Only one study focused on PNS of the auriculotemporal nerve (ATN) connecting the facial and trigeminal nerves, but the data were insufficient to calculate the frequency of PNS [32]. In a study looking at the PNS of skull base tumors in the PPF, intracranial spread was found in 32% of cases [31].

In general, studies are insufficient to allow for conclusive statements about the incidence of PNS of all CNs with regard to MRI data and histology.

### 4.5. MRI Technique and Protocols

Hanna et al. reported using a high-resolution protocol to detect PNS on MRI, but the parameters of the MRI protocol were incomplete [16]. Using small FOV, thin collimation and a high-resolution matrix covering all considered CNs with the recommended scanning area was defined as a prerequisite for obtaining high-resolution images in MRI neurography of the large nerve [20]. Despite the fact that a control study of MRI neurography between the 1.5 T and 3 T scanners was not conducted, the reduced time of studies and the appropriate sequences made the latter optimal to perform [7,36].

All studies were conducted using contrast-enhanced MRI. The slice thickness for the targeted MRI was mainly 2 mm. A study with conventional MRI protocols used 3 mm, 4 mm, or 5 mm of slice thickness, which did not affect the sensitivity or specificity of the study [19]. FOV in both protocols ranged from 18 to 25 cm (Table S3).

Fat-suppressed postcontrast T1 [36,39] was used for both protocols in axial and/or coronal views. T1-weighted postcontrast sequence with FS better defined the borders of the enhancing lesion at the fat-containing background due to the suppression of the signal of fat [16,29,30,31,40]. Moreover, it was useful to detect tumor extension to the PPF, skull base, and oral and maxillofacial regions [30,31] to assess the entire trigeminal nerve [29]. Nemzek et al. used a T1-weighted sequence with fat saturation to better reveal the contrast enhancement of the infraorbital nerve, but magnetic susceptibility artifacts could lead to a quality degradation [22]. Postcontrast T1-weighted sequences without FS were recommended when fat suppression artifacts from FS sequences interfered with interpretation [29].

Baulch et al. [21] applied 3D T1 fat sat postcontrast sequences with 1 mm slice thickness for MRI neurography: spoiled gradient-recalled (SPGR) to obtain high soft tissue contrast compared with T1 spin echo (SE) sequence [41], and sagittal magnetization-prepared rapid gradient echo (Mprage), which allows for reconstruction in different planes without loss of image resolution [20,21] (Table S4). Sagittal postcontrast T1-weighted FS sequences have been obtained in two studies [19,31] and are considered useful for fifth nerve evaluation, along with axial MRI images [42].

Two studies showed that precontrast axial T1-weighted sequences were superior to depict PNS to PPF and other skull base fat spaces [8]; moreover, they were highly effective in all cases of early tumor spread in PPF followed by T1 postcontrast (92%) and T2 (56%)-weighted images [31]. T2-weighted images better visualized the tumor and inflammatory changes [31]. Fat-suppressed coronal T2-weighted sequences have been performed in MRI neurography protocols [20,21] to detect denervation changes in the muscles of mastication and facial expression [43].

Overall, fat-suppressed postcontrast T1 was the sequence of choice for conventional and MRI neurography protocols. Large nerve PNS should be evaluated using high-resolution MRI neurography. Further studies of high-resolution MRI protocols are needed to assess the PNS of other CNs and to compare different MRI protocols.

MRI neurography with 3T MRI using high-resolution protocols better visualized the superficial branches of the large nerve and predicted its anatomical extent [13].

New MRI techniques, such as 3D magnetic resonance neurography and black blood MRI, may offer a more detailed evaluation of CNs’ peripheral branches. This could lead to improved detection of PNS in the early stages. Additionally, diffusion tensor tractography could be useful for studying the trajectory of CNs [9,13,44,45].

### 4.6. MRI Features of PNS

All studies reported nerve enhancement and enlargement to determine PNS. Only in the study by Chang et al. [8] were MRI features compared with respect to tumor histology in eight patients, and the most common features were enlargement and enhancement of nerves (100%), followed by a mass in Meckel’s cave (88%) or in the cavernous sinus (75%), as well as denervation changes in the muscles (63%).

Seven studies have used perineural fat obliteration to define PNS [16,19,20,21,22,31,32]. Nader et al. [19] described enhancement and/or fat replacement of the SMF as an MRI feature of intratemporal facial nerve PNS.

Neurography studies to describe the large nerve PNS to the three aforementioned MRI features applied secondary denervation changes in the muscles of facial expression or mastication [20,21,43] (Table S3), which are indicated in the literature as an indirect sign [36]. However, studies have not compared the MRI patterns of denervated muscles depending on the chronicity of the process with histological data [8,20,21,29].

Other studies using conventional MRI included expansion and/or erosion (with contrast enhancement) of the skull base foramina [8,22,29,30], enhancement of a mass in the Meckel’s cave/gasserian ganglion area, lateral bulging of the cavernous sinus dural membranes [8,22,29,42], and changes of the signal intensity of the trigeminal cistern on T1 and T2-WI [29] to define PNS.

Replacement of fat in PPF and in skull base foramina has been defined as a sign of pathology [46,47,48]. MRI depicted tumor in bony canals and foramina that connect to PPF, especially in the sphenopalatine foramen (57%) and inferior orbital fissure (57%), and in 60% of cases, bony abnormalities (erosion or destruction) were observed as tumor replacement of hyperintensity of bone marrow [31].

### 4.7. Strengths and Limitations

This study is based on a restricted number of retrospective studies with some studies subject to sampling bias. Other limitations include different MRI devices and protocols, incomplete radiological information, and lack of the interobserver agreement in individual studies regarding MRI features of the PNS. One study [28] was not designed to assess the diagnostic accuracy of MRI, and another had low-quality MR images [29]. However, the strength of our study lies in the fact that the current systematic review and meta-analysis fills an existing knowledge gap in the available literature regarding the diagnostic accuracy of MRI and outlines the difficulty of detecting PNS in head and neck tumors with MRI.

## 5. Conclusions

The precise detection of PNS is vital because it influences treatment approaches and prognosis in head and neck tumors. The presented systematic review and meta-analysis showed that MRI was highly accurate in depicting the perineural spread of CNs, yet this statement was based on scarce and heterogenous evidence. Nerve enhancement and enlargement were the most common MRI features of the PNS. A literature review verified that the most common histological tumor types afflicted by PNS were SCC and ACC. The most commonly involved nerves in PNS were the facial and trigeminal nerves, with the most frequently affected division of the latter being the maxillary nerve (V2). Prospective studies in larger samples should be conducted in the future for unbiased assessment of diagnostic accuracy and for unraveling diagnostic variables that distinguish PNS from other neoplastic and non-neoplastic diseases of the cranial nerves. A potential clinical and patient management implication is that clinicians can rely on this highly accurate imaging method when the clinical suspicion of PNS arises. At the same time, radiologists should carefully look for subtle signs of PNS when reviewing head and neck MRI.

## Figures and Tables

**Table 1:** Summary of the studies included in the meta-analysis.

Author	Year	N of Patients	Mean Age	N of CNs	TP	TN	FP	FN	Se	Sp	PPV	NPV
Hanna et al. [16]	2007	26	**-**	27	14	11	2	0	1.00	0.85	0.88	1.00
Nader et al. [19] (DFN model 1)	2019	52	58	52	8	36	5	3	0.73	0.88	0.62	0.92
Nader et al. [19] (DFN model 2)	2019	52	58	52	5	41	0	6	0.46	1.00	1.00	0.87
Nader et al. [19] (SMF)	2019	49	58	49	16	24	5	4	0.8	0.83	0.76	0.86
Baulch et al. [21]	2015	33	62	57	36	16	3	2	0.95	0.84	0.92	0.75
Gandhi et al. [20]	2011	25	59	48	30	17	1	0	1.00	0.94	0.97	1.00
Warren et al. [28]	2016	48	60	48	46	0	0	2	0.96	-	1.00	-
Chang et al. [8]	2004	8	58	8	8	0	0	0	1.00	-	1.00	-
Nemzek et al. [22]	1998	19	55	45	18	0	0	1	0.95	-	1.00	-
Schmalfuss et al. [32]	2002	7	62	7	7	0	0	0	1.00	-	1.00	-
Majoie et al. [29]	1997	2	57	2	2	0	0	0	1.00	-	1.00	-
Shimamoto et al. [30]	2012	13	58	13	8	2	0	3	0.73	1.00	1.00	0.4
Tomura et al. [31]	1999	12	-	12	12	0	0	0	1.00	-	1.00	-

Abbreviations: CNs, cranial nerves; TP, true positive; TN, true negative; FP, false positive; FN, false negative; Se, sensitivity; Sp, specificity; PPV, positive predictive value; NPV, positive predictive value; DFN, descending facial nerve; SMF, stylomastoid foramen.

## Data Availability

All data generated or analyzed during this study are included in this published article (and its Supplementary Materials).

## Supplementary Materials

The following supporting information can be downloaded at https://www.mdpi.com/article/10.3390/diagnostics14010113/s1, Checklist S1: PRISMA 2020 Checklist. Table S1: QUADAS-2 assessments. Table S2: Summary of cranial nerves afflicted by PNS, histology, and site of tumor origin. Table S3: Summary of MRI vendors and MRI protocols in the studies. Table S4: MRI protocols used in the studies. Table S5: Distribution of the trigeminal and facial nerves afflicted by PNS in the studies. Table S6: Summary of histological forms of head and neck tumors afflicted by PNS. Table S7: Database search strategy. Table S8: QUADAS-2 domains and questions.
