# Supplementary material for: Diagnostic Accuracy of MRI in Detecting the Perineural Spread of Head and Neck Tumors: A Systematic Review and Meta-Analysis
Source: Diagnostics (Basel). 2024 Jan 4;14(1):113. doi: 10.3390/diagnostics14010113 (PMC10795679; doi:10.3390/diagnostics14010113)
Supplement: Supplementary file 1 [file diagnostics-14-00113-s001.zip › Table S1 QUADAS-2 assessments.pdf]

Table S1. QUADAS-2 assessments.

	Risk of bias				Applicability concern		
**First author**	**Patient selection**	**Index test**	**Reference standard**	**Flow and timing**	**Patient selection**	**Index test**	**Reference standard**
Hanna et al.	Low	Low	Low	Low	Low	Unclear	Low
Nader et al.	High	Low	Low	Low	Low	Unclear	Low
Baulch et al.	High	Low	Low	Low	Low	Low	Low
Gandhi et al.	High	Low	Low	Low	Low	Low	Low
Warren et al.	Low	High	Low	Low	Low	High	Low
Chang et al.	High	Low	Low	Low	Low	unclear	Low
Nemzek et al.	High	Low	Low	Low	Low	Unclear	Low
Schmalfuss et al.	High	Low	unclear	Low	Low	High	Low
Majoie et al.	High	Low	unclear	Low	Low	unclear	Low
Shimamoto et al.	High	Low	Low	Low	Low	Low	Low
Tomura et al.	Low	Low	unclear	Low	Low	Low	Low
