# Supplementary material for: Diagnostic Accuracy of MRI in Detecting the Perineural Spread of Head and Neck Tumors: A Systematic Review and Meta-Analysis
Source: Diagnostics (Basel). 2024 Jan 4;14(1):113. doi: 10.3390/diagnostics14010113 (PMC10795679; doi:10.3390/diagnostics14010113)
Supplement: Supplementary file 1 [file diagnostics-14-00113-s001.zip › Table S2 Summary of cranial nerves afflicted by PNS, site of tumor origin and histology..pdf]

Table S2. Summary of cranial nerves afflicted by PNS, site of tumor origin and histology.

Author	Cranial nerves examined for PNS	Cranial nerves afflicted by PNS	Tumor Histology	Site of tumor origin
Hanna et al.	-	-	ACC	PG, sinuses, NP
Nader et al. (DFN model 1)	ITFN (DFN segment)	8 DFN	ACC, SCC, salivary ductal carcinoma, acinic cell carcinoma, adenocarcinoma, MEC, carcinosarcoma, oncocytic carcinoma, BCC with squamous differentiation	-
Nader et al. (DFN model 2)	ITFN (DFN segment)	5 DFN	ACC, SCC, salivary ductal carcinoma, acinic cell carcinoma, adenocarcinoma, MEC, carcinosarcoma, oncocytic carcinoma, BCC with squamous differentiation	-
Nader et al. (SMF)	ITFN (SMF segment)	16 SMF	ACC, SCC, salivary ductal carcinoma, acinic cell carcinoma, adenocarcinoma, MEC, carcinosarcoma, oncocytic carcinoma, BCC with squamous differentiation	-
Baulch et al.	9 V1, 21 V2, 14 V3, 13 VII, 1 ATN	8 V1, 12 V2, 9 V3, 9 VII	SCC, melanoma, ACC, salivary gland tumor	-
Gandhi et al.	8 V1, 21 V2, 11 V3, 8 VII	5 V1, 13 V2, 5 V3, 7 VII	SCC, BCC, desmoplastic melanoma, poorly differentiated adenocarcinoma	-
Warren et al.	V, VII	12 V3 and VII, 18 VII, 18 V1, 21 V2, 14 V3, 2 great auricular nerves	cutaneous SCC	unknown origin (28%), cheek (20%), nose (16%), temple (14%), forehead (8%), preauricular (8%), ear (2%), eyelid (2%), postauricular (2%)
Chang et al.	-	4 V1, 4 V2, 5 V3, 3 III, 2 IV, 4 VI, 4 VII, 1 VIII, 1 IX, 1 X, 1 XI, 1 XII	Melanoma (5 desmoplastic, 2 mucosal, 1 poorly differentiated spindle-cell melanotic tumor)	forehead (12.5), nose (12.5), NP (12.5%), lower (25%) and upper (12.5%) lip, supraorbital skin (12.5%), infraorbital skin (12.5%)
Nemzek et al.	-	16 V (5 in Meckel's cave, 7 in the cavernous sinus, 1 V1, 6 V2, 5 V3), 4 ION, 3 IV, 3 XII, 4 III, 4 VI, 1 II, 2 VII, 2 IX, 2 X, 2 XI	ACC, SCC, poorly differentiated carcinoma, salivary duct carcinoma, MEC, chordoma, meningioma	NP (26%), PG (16%), nasal cavity (11%), maxillary sinus (11%), RMT (11%), clivus (5%), middle fossa (5%), eyelid inferior (5%), ethmoid (5%), forehead skin (5%)

Schmalfuss et al.	VII, ATN, V3, trigeminal cistern rootlets and trigeminal ganglion	5 V3, 1 V2, 7 ATN, 6 VII	ACC, adenocarcinoma, SCC, MEC, metastatic undifferentiated carcinoma of unknown primary site combined with lymphoma, malignant schwannoma, neurofibroma, tumor of unknown pathologic origin	PG, skin, unknown primary site
Majoie et al.	V	1V2, 1 V1	malignant melanoma cheek, Non-Hodgkin lymphoma	cheek, orbit
Shimamoto et al.	-	-	ACC	palate (45%), sublingual gland (18%), lip (9%), cheek (9%), retromolar region (9%), angle of mandible (9%)
Tomura et al.	PPF obliteration	infratemporal fossa, sphenopalatine foramen, sphenoid sinus, greater palatine canal, inferior orbital fissure, pterygoid canal, foramen rotundum	SCC, NP angiofibroma	maxillary sinus (83.4%), NP (8.3%), gingiva (8.3%)

Notes

"-" signifies missing data

ITFN - intratemporal facial nerve

DFN - descending facial nerve

SMF - stylomastoid foramen

ION - infraorbital nerve

PPF - pterygopalatine fossa

ATN - auriculotemporal nerve

MEC PG - mucoepidermoid carcinoma parotid gland

RMT - retromolar trigone

RLN - recurrent laryngeal nerve

ACC -adenoid cystic carcinoma

SCC - squamous cell carcinoma

BCC - basal cell carcinoma
