# Supplementary material for: Diagnostic Accuracy of MRI in Detecting the Perineural Spread of Head and Neck Tumors: A Systematic Review and Meta-Analysis
Source: Diagnostics (Basel). 2024 Jan 4;14(1):113. doi: 10.3390/diagnostics14010113 (PMC10795679; doi:10.3390/diagnostics14010113)
Supplement: Supplementary file 1 [file diagnostics-14-00113-s001.zip › Table S3 Summary of MRI vendors and MRI protocols in the studies.pdf]

Table S3. Summary of MRI vendors and MRI protocols in the studies.

First author	Manufacturer & model	Field strength	Coil	Contrast injection, agent	MRI protocols	MRI sequences	Slice thickness (mm)	gap (mm)	FOV (cm)	Imaging features of PNS
Hanna et al.	-	-	head and neck	-	conventional	axial, coronal and sagittal T2 and T1 precontrast, axial T1 postcontrast with FS	-	-	-	Replacement of normal perineural fat with tumor, enhancement with gadolinium (regardless of size), and increased size of the nerve in question (regardless of enhancement)
Nader et al.	-	-	-	-	conventional	axial T2, T1 precontrast, axial, coronal and sagittal T1 FS postcontrast	3-5	-	-	Enhancement and asymmetry of the DFN, abnormal enhancement and/or loss of the normal T1 fat-related signal hyperintensity
Baulch et al.	Siemens Magneton Skyra system	3T	64 Channel Head/Neck	-	Targeted MRI (neurogram)	axial T2 FS, axial, coronal T1 precontrast, coronal T1 FS postcontrast, coronal T2 SPACE (3D), sagittal T1 Mprage (3D) postcontrast	2	0.4 and 0.5	18 (axial T2 FS, axial, coronal T1 pre-, coronal T1 FS postgad), 20 (T2 SPACE (3D)), 24 (T1 Mprage (3D) postgad)	asymmetrical thickening or enhancement with obliteration of perineural fat pads, ± secondary denervation changes in the muscles of facial expression or mastication
	GE 3T Discovery MR750	3T	64 Channel Head/Neck	-	Targeted MRI (neurogram)	axial and coronal T1 FSE, coronal T2 fat sat, 3D T1 fat sat contrast spoiled gradient echo (SPGR), coronal T1 fat sat	2mm for precontrast and 1mm for	1	16 (axial T1 FSE), 18 (all other sequences)	

							postcontrast SPGR images			
Gandhi et al.	Signa Echospeed system (GE Medical Systems)	1,5 T	phased array surface (temporal lobe surface coil)	-	Targeted MRI (neurogram)	coronal T2 FS, axial and coronal T1 precontrast, axial and coronal T1 FS postcontrast	2	0.5	18	asymmetrical thickening of a nerve, asymmetrical enhancement of a nerve, secondary denervation changes in the muscles of mastication and facial expression, and obliteration of perineural fat pads
	Avanto system (Siemens, Germany)	1,5 T	standard head	-	Targeted MRI (neurogram)	axial T2 FS, axial and coronal T1 precontrast, coronal T1 FS postcontrast	2	1	20	
Warren et al.	-	3T	-	-	Targeted MRI (neurogram)	-	-	-	-	-
Chang et al.	-	-	-	-	conventional	sagittal, axial, and coronal T1, axial T2 FSE with FS, axial and coronal T1 postcontrast with FS	-	-	-	abnormal nerve thickening and/or contrast enhancement, expansion and/or erosion of skull base foramina, enhancing mass in Meckel's cave, lateral bulging of the cavernous sinus dura, and denervation atrophy of the muscles of mastication
Nemzek et al.	-	1.5 and 0.3T **(1 patient)**	-	gadopentetate dimeglumine (0.1 mmol/ kg)	conventional	sagittal, axial T1- and T2-WI precontrast, axial and coronal T1-WI postcontrast with fat saturation	3-5 (for T1 WI)	-	-	enhancement or enlargement of the neural foramen, enhancement of the Meckel's cave, replacement of normal fat within neural foramina, enlargement of the nerve or cavernous sinus, or enhancement of the individual nerve
Schmalfuss et al.	-	-	-	-	-	-	-	-	-	enlargement or abnormal enhancement of the evaluated nerves or obliteration of the perineural fat pad or both

Majoie et al.	-	-	-	gadopentetate dimeglumine (0.1 mmol/kg)	conventional	axial T2 SE and PD SE, coronal, axial TI SE precontrast, coronal, axial TI SE postcontrast with and without FS	3mm (for T1 SE pre- and postco ntrast sequen ces)	-	-	abnormal nerve thickening with enhancement; concentric expansion of the skull base foramina and extracranial nerve canals; replacement of the normal trigeminal cistern hypointensity on T1-weighted images or hyperintensity on T2-weighted images by an isointense mass or enhancement of a mass in the Meckel's cave/gasserian ganglion area; lateral bulging of the cavernous sinus dural membranes and denervation atrophy of the masticator muscles
Shimamoto et al.	Signa LX, GE	1,5T	head	Magnevist, Omniscan, Prohance (0.2 ml kg–1)	conventional	axial T1 and T2 FSE without or with chemical shift selective FS, postcontrast T1 (axial, cor) with chemical shift selective FS	5	1	25x25	abnormal signal intensity, contrast enhancement or widening of the pterygopalatine fossa, palatine foramen, incisive canal, mandibular foramen and mandibular canal, and enlargement or excessive contrast enhancement of a nerve
Tomura et al	Signa or Horizon; GE Medical Systems	1,5T	-	gadopentetate dimeglumine (Gd-DTPA; Schering, AG),	conventional	axial T2 SE and PD SE, axial T2 FSE and PD FSE, cor, axial, sagittal T1 precontrast, cor, axial T1 postcontrast with and without fatsat, sagittal T1 postcontrast	3-5	1, 2	18-22	Obliteration of fat within the pterygopalatine fossa

Notes

"-" signifies missing data
FS - fat-supressed
FSE - fast spin echo
DFN - descending facial nerve
SMF - stylomastoid foramen
