# Supplementary material for: Diagnostic Accuracy of MRI in Detecting the Perineural Spread of Head and Neck Tumors: A Systematic Review and Meta-Analysis
Source: Diagnostics (Basel). 2024 Jan 4;14(1):113. doi: 10.3390/diagnostics14010113 (PMC10795679; doi:10.3390/diagnostics14010113)
Supplement: Supplementary file 1 [file diagnostics-14-00113-s001.zip › Table S4 MRI protocols used in the studies.pdf]

Table S4. MRI protocols used in the studies.

**Total**<br>**n = 11**	**Sequences**	**slice thickness, TR, TE**
**Conventional protocols,**<br>**n= 7**		
**1. Hanna et al.**	axial T2	-
	coronal T2	-
	sagittal T2	-
	axial T1 precontrast	-
	axial T1 postcontrast with FS	-
**2. Tomura et al.**	axial T2 SE	3-5 mm, 1800-3000 ms, 80-100 ms
	axial PD SE	3-5 mm, 1800-3000 ms, 30-40 ms
	axial T2 FSE	3-5 mm, 3800-4200 ms, 100-112 ms
	axial PD FSE	3-5 mm, 3800-4200 ms, 100-112 ms
	axial T1	3-5mm, 300-680ms, 11-22 ms
	coronal T1	3-5mm, 300-680ms, 11-12 ms
	axial T1 postcontrast with and without FS	3-5mm, -
	coronal T1 postcontrast with and without FS	3-5mm, -
	sagittal T1 pre- and postcontrast	3-5mm, -
**3. Nemzek et al.**	axial T1 precontrast	3-5 mm, -
	sagittal T1 precontrast	3-5 mm, -
	axial T2	-
	sagittal T2	-
	axial T1 postcontrast with fat saturation	3-5 mm, -
	coronal T1 postcontrast with fat saturation	3-5 mm, -
**4. Chang et al.**	axial T2 FSE with FS	-
	axial T1 precontrast	-
	sagittal T1 precontrast	-
	coronal T1 precontrast	-
	axial T1 postcontrast with FS	600 ms, 11 ms
	coronal T1 postcontrast with FS	600 ms, 11 ms
**5. Nader et al.**	axial T2	3-5 mm, -

	axial T1 precontrast	3-5 mm, -
	axial T1 FS postcontrast	3-5 mm, -
	coronal T1 FS postcontrast	3-5 mm, -
	sagittal T1 FS postcontrast	3-5 mm, -
**6. Shimamoto et al.**	axial T2 FSE with or without chemical shift selective FS	5mm, 3500ms, 98 ms
	axial T1 precontrast SE without or with chemical shift selective FS	5mm, 400-500ms, 9-10 ms
	axial T1 postcontrast SE with chemical shift selective FS	5mm, 400-500ms, 9-10 ms
	coronal T1 postcontrast SE with chemical shift selective FS	5mm, -
**7. Majoie et al.**	axial PD SE	-
	axial T2 SE	-
	axial TI SE precontrast	3 mm, -
	coronal TI SE precontrast	3 mm, -
	axial TI SE postcontrast with and without FS	3 mm, -
	coronal TI SE postcontrast with and without FS	3 mm, -
**Targeted MRI (neurography) protocols, n=4 (33 %)**	**Sequences**	**slice thickness, TR, TE**
**1. Gandhi et al. (GE) 1.5T**	coronal T2 FS	2 mm, 6800 ms, 102 ms
	axial T1 precontrast	2 mm, 680 ms, Min full
	coronal T1 precontrast	2 mm, 680 ms, Min full
	axial T1 FS post contrast	2 mm, 675 ms, Min full
	coronal T1 FS post contrast	2 mm, 675 ms, Min full
**2. Gandhi et al. (Siemens) 1.5T**	axial T2 FS	2 mm, 3300 ms, 99 ms
	axial T1 precontrast	2 mm, 620 ms, 15 ms
	coronal T1 precontrast	2 mm, 620 ms, 15 ms
	coronal T1 FS post contrast	2 mm, 581 ms, 13 ms
**3. Baulch et al. (Siemens) 3T**	axial T2 FS	2 mm, 4400 ms, 89 ms
	axial T1 precontrast	2 mm, 798 ms, 9.1 ms
	coronal T1 precontrast	2 mm, 750 ms, 9.1 ms
	coronal T1 FS postcontrast	2 mm, 986 ms, 9.1 ms
	coronal T2 3D SPACE	- , 1000 ms, 136 ms
	sagittal T1 Mprage (3D) postcontrast	- , 2300 ms, 2.29 ms
**4. Baulch et al. (GE) 3T**	axial T1 FSE precontrast	2 mm, 808 ms, min full
	coronal T1 FSE precontrast	2 mm, 711 ms, min full
	coronal T2 fat sat	2 mm, 7015 ms, 85 ms

	coronal T1 fat sat	2 mm, 683 ms, min full ms
	3D T1 fat sat contrast spoiled gradient echo (SPGR)	1 mm, 6.6 ms, minimum

Notes:
"-" signifies missing data
TE - echo time, TR - repetition time, TSE - turbo spin echo, FSE – fast spin echo, FS – fat suppression, PD – proton density, 3D SPACE - three-dimensional sampling perfection with application-optimized contrasts by using different flip angle evolutions, Mprage - magnetization-prepared rapid gradient echo, SPGR - spoiled gradient-recalled (SPGR) sequence
