# Supplementary material for: Diagnostic Accuracy of MRI in Detecting the Perineural Spread of Head and Neck Tumors: A Systematic Review and Meta-Analysis
Source: Diagnostics (Basel). 2024 Jan 4;14(1):113. doi: 10.3390/diagnostics14010113 (PMC10795679; doi:10.3390/diagnostics14010113)
Supplement: Supplementary file 1 [file diagnostics-14-00113-s001.zip › Table S5 Distribution of trigeminal and facial nerves afflicted by PNS in the studies.pdf]

Table S5. Distribution of trigeminal and facial nerves afflicted by PNS in the studies.

**Authors**	**V1**	**V2**	**V3**	**VII**
Gandhi et al.	5	13	5	7
Warren et al.	18	21	14	18
Baulch et al.	8	12	9	9
Chang et al.	4	4	5	4
Nemzek et al.	1	6	5	2
Schmalfuss et al.	-	1	5	6
Majoie et al.	1	1	-	-
Nader et al (ITFN)	-	-	-	24
**Total**	**37**	**58**	**43**	**70**

Notes:

"-" signifies missing data

V1 – ophthalmic division of the trigeminal nerve

V2 – maxillary division of the trigeminal nerve

V3 - mandibular division of the trigeminal nerve

ITFN - intratemporal facial nerve
