# Supplementary material for: Diagnostic Accuracy of MRI in Detecting the Perineural Spread of Head and Neck Tumors: A Systematic Review and Meta-Analysis
Source: Diagnostics (Basel). 2024 Jan 4;14(1):113. doi: 10.3390/diagnostics14010113 (PMC10795679; doi:10.3390/diagnostics14010113)
Supplement: Supplementary file 1 [file diagnostics-14-00113-s001.zip › Table S6 Summary of histological forms of head and neck tumors afflicted by PNS.pdf]

Table S6 – Summary of histological forms of head and neck tumors afflicted by PNS

**First author**	**Nader et al.**	**Baulch et al.**	**Gandhi et al.**	**Nemzek et al.**	**Tomura et al.**	**Schmalfuss et al.**	**Hanna et al.**	**Warren et al.**	**Chang et al.**	**Shimamoto et al.**	**Majoie et al.**	**Summary**
**Patients' number with PNS who had reference standard test**	**21 (out of 58)**	**33**	**25**	**19**	**12**	**7 (out of 15)**	**26**	**48**	**8**	**11 (out of 13)**	**2 (out of 15)**	**171**
**Tumor histology**												
SCC	9 (42.9%)	27(81.8%)	22(88%)	10(52.6%)	11(91.7%)	2(28.6%)		48(100%)***				**129**
ACC	3(14.3%)	2(6.1%)		4(21%)		2(28.6%)	26(100%)			11(100%)		**48**
Melanoma		3(9.1%)	1(4%)#						8(100%)		1(50%)	**13**
Mucoepidermoid carcinoma	1 (4.8%)			1(5.3%)		1(14.3%)						**3**
Salivary ductal carcinoma	3(14.3%)			1(5.3%)								**4**
Salivary gland tumor		1(3%)										**1**
nasopharyngeal angiofibroma					1(8.3%)							**1**
BCC			1(4%)									**1**
BCC with squamous differentiation	1 (4.8%)											**1**
small round cell neoplasm												
Lymphoma											1(50%)	**1**

schwannoma						1**(14.3%)						**1**
Acinic cell carcinoma	1 (4.8%)											**1**
Adenocarcinoma	1 (4.8%)											**1**
Carcinosarcoma	1 (4.8%)											**1**
Oncocytic carcinoma	1 (4.8%)											**1**
Poorly differentiated carcinoma			1(4%)	1(5.3%)								**2**
meningioma (atypical)				1(5.3%)								**1**
chordoma				1(5.3%)								**1**
unknown						1(14.3%)						**1**
**Notes:**												
* - trigeminal schwannoma	*** - cutaneous SCC											
** - malignant schwannoma	# - desmoplastic melanoma											
SCC - squamous cell carcinoma												

ACC -adenoid cystic carcinoma												
BCC - basal cell carcinoma												
