# Supplementary material for: Diagnostic Accuracy of MRI in Detecting the Perineural Spread of Head and Neck Tumors: A Systematic Review and Meta-Analysis
Source: Diagnostics (Basel). 2024 Jan 4;14(1):113. doi: 10.3390/diagnostics14010113 (PMC10795679; doi:10.3390/diagnostics14010113)
Supplement: Supplementary file 1 [file diagnostics-14-00113-s001.zip › Table S7 Database Search strategy.pdf]

Database search strategy

	**Key words and operators**	**Combination of key words**
1	perineural spread	
2	MRI	
3	perineural invasion	
4	CT	
5	computed tomography	
6	FDG PET or PET or 18F-fluorodeoxyglucose PET	
7	retrospective analysis	
8	MRI OR CT*	
9	pathologic correlation	
10	radiologic-pathologic correlation	
11	magnetic resonance imaging	
12	perineural tumor spread	
13	perineural tumor invasion	
14	head and neck tumors	
15	head and neck cancer	
	AND/1-2	perineural spread And MRI<br>"perineural spread" And MRI
	AND/3,2	perineural invasion And MRI<br>"perineural invasion" And MRI
	AND/1,4	perineural spread And CT<br>“perineural spread” And CT
	AND/1,6	"perineural spread" And FDG PET<br>perineural spread And FDG PET<br>"perineural spread" And PET<br>perineural spread And 18F-fluorodeoxyglucose PET
	AND/1 ,7	perineural spread And retrospective analysis
	AND/1 ,8	"perineural spread" AND (MRI OR CT*)
	AND/3 ,9	perineural invasion And pathology correlation
	AND/1, 10	perineural spread And radiologic-pathologic correlation
	AND/1 ,11	perineural spread And magnetic resonance imaging
	AND/1 ,5	perineural spread And computed tomography
	OR/AND 12,2,1,6	(perineural tumour spread OR MRI) AND (perineural spread OR PET)
	OR/AND 13, 2, 1, 6	(perineural tumor invasion OR MRI) AND (perineural spread OR PET)
	OR/AND 12, 2, 1, 4	(perineural tumour spread OR MRI) AND (perineural spread OR CT)
	OR/AND 12,2,1,9	(perineural tumour spread OR MRI) AND (perineural spread OR pathologic correlation)
	AND 9, 14	pathologic correlation AND head and neck tumors
	OR 1,15, 4, 2	[perineural spread] OR [head and neck cancer] OR [CT] OR [MRI]
	AND	[18F-FDG PET], [perineural spread], [head and neck cancer], [CT], and [MRI]
