# Supplementary material for: Diagnostic Accuracy of MRI in Detecting the Perineural Spread of Head and Neck Tumors: A Systematic Review and Meta-Analysis
Source: Diagnostics (Basel). 2024 Jan 4;14(1):113. doi: 10.3390/diagnostics14010113 (PMC10795679; doi:10.3390/diagnostics14010113)
Supplement: Supplementary file 1 [file diagnostics-14-00113-s001.zip › Table S8 QUADAS-2 domains and questions.pdf]

Results of the assessment of various domains and scoring system of the QUADAS-2 tool

1	**RISK OF BIAS**<br>Concerns about risk of bias were rated as “yes,” “no” or “unclear.” The “unclear” category was used when incomplete data were reported. “Yes” indicates low risk of bias, and “no” or “unclear” indicates high risk of bias
1a	***Patient selection***
	Could the selection of patients have introduced bias?
	*Was a consecutive sample enrolled?
	* Was the selection method of patients reported?
	consecutive – yes (Low bias), no information – no (High bias)
1b	***Index test***
	Could the conduct or interpretation of the index test introduce bias?
	*Did all studies report MRI features of perineural spread of head and neck tumors?
	If reported – yes (Low), not reported -no (High)
1c	***Reference standard***
	Could the reference standard or its conduct or interpretation have introduced bias?
	*Which reference method was used?<br>*Did all patients undergo the reference method?
	histology and/or surgery, complete reference method – yes (low); histology and/or surgery, incomplete reference method – unclear (unclear)
1d	***Flow and timing***
	Could the patient flow have introduced bias?
	*Was there an appropriate interval between the index test and reference standard?
	– **yes** for all studies **(low)**
2	**APPLICABILITY CONCERN**<br>Concerns about applicability were rated as “low,” “high,” or “unclear.” The “unclear” category was used only when insufficient data are reported.
2a	***Patient selection***
	Whether the histology of all tumors with perineural spread included in the studies:
	- if included - LOW, not included -HIGH
2b	***Index test***
	Did all studies report data on MRI protocols:
	– yes – LOW, no – High, incomplete - unclear
2c	***Reference standard***
	Whether pathologists were blinded or not:<br>- LOW for all studies

Results of the assessment of various domains of the QUADAS-2 tool

	Risk of bias				Applicability concern		
**First author**	**Patient selection**	**Index test**	**Reference standard**	**Flow and timing**	**Patient selection**	**Index test**	**Reference standard**
Hanna et al.	yes	yes	yes	yes	Low	unclear	Low
Nader et al.	no	yes	yes	yes	Low	unclear	Low
Baulch et al.	no	yes	yes	yes	Low	Low	Low
Gandhi et al.	no	yes	yes	yes	Low	Low	Low
Warren et al.	yes	no	yes	yes	Low	High	Low
Chang et al.	no	yes	yes	yes	Low	unclear	Low
Nemzek et al.	no	yes	yes	yes	Low	unclear	Low
Schmalfuss et al.	no	yes	unclear	yes	Low	High	Low
Majoie et al.	no	yes	unclear	yes	Low	unclear	Low
Shimamoto et al.	no	yes	yes	yes	Low	Low	Low
Tomura et al.	yes	yes	unclear	yes	Low	Low	Low
